# Improved Clinical Outcomes With Elexacaftor/Tezacaftor/Ivacaftor in Patients With Cystic Fibrosis and Advanced Lung Disease: Real‐World Evidence From an Italian Single‐Center Study

**DOI:** 10.1002/prp2.70083

**Published:** 2025-04-02

**Authors:** Nicola Perrotta, Luigi Angelo Fiorito, Gianfranco Casini, Rossella Gentile, Roberta Vescovo, Alfonso Piciocchi, Roberta Lobello, Carlo Cappelli, Roberto Poscia, Giuseppe Cimino

**Affiliations:** ^1^ Department of Physiology and Pharmacology “V. Erspamer” Sapienza University Rome Italy; ^2^ Pharmacy Unit AOU Policlinico Umberto I‐Sapienza University Rome Italy; ^3^ Biostatistics Unit GIMEMA Foundation Rome Italy; ^4^ Cystic Fibrosis Center AOU Policlinico Umberto I‐Sapienza University Rome Italy; ^5^ Clinical Research Unit AOU Policlinico Umberto I‐ Sapienza University Rome Italy; ^6^ Interdepartmental Center for Rare Diseases AOU Policlinico Umberto I‐ Sapienza University Rome Italy

**Keywords:** CFTR, cystic fibrosis, effectiveness, Elexacaftor‐Tezacaftor‐Ivacaftor, safety

## Abstract

The combination of Elexacaftor/Tezacaftor/Ivacaftor (ETI) has resulted in a significant improvement in lung function and global clinical parameters, which have not been previously achieved with other CFTR modulators. However, there is a paucity of evidence in the literature on the long‐term use of ETI in adolescents and patients with severe pulmonary impairment. Furthermore, the response to ETI may differ between homozygotes and heterozygotes, as well as between naïve patients and those previously treated with other CFTR modulators. A retrospective study was conducted to examine changes in percent predicted forced expiratory volume in 1 s (ppFEV_1_), body‐mass index (BMI), and sweat chloride concentration (SwCl) at baseline and at 6, 12 and 24 months after the initiation of ETI. Secondary outcomes included the number of pulmonary exacerbations, Cystic Fibrosis Questionnaire‐Revised (CFQ‐R) score, adverse events, mortality and transplantation rates. 139 subjects were included and followed up for up to 2 years after starting ETI. The results demonstrated a significant improvement in ppFEV_1_ and BMI after 12 months of therapy (respectively, 16%, *p* < 0.001; +1.5 kg/m^2^, *p* = 0.005), with a slight decline in the values after 24 months. This effect was independent of genotype and showed a different degree of response in naïve subjects compared to patients previously treated with other CFTR modulators. SwCl decreased from 84 to 37 mmol/L over 24 months (*p* < 0.001). 58.3% reduction of PEx rate was observed compared to the number of exacerbations prior to ETI. Overall, lung function, SwCl, PEx rate, CFQ‐R scores and BMI improved after 24 months of ETI treatment. ETI was well tolerated, and none of the patients interrupted the treatment due to toxicity.

## Graphical abstract

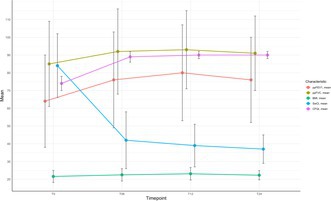

**Abbreviations**
ADRs: adverse drug reactions
CFQ‐R: Cystic Fibrosis Questionnaire‐Revised
CFTR: cystic fibrosis transmembrane conductance regulator
ETI: Elexacaftor‐Tezacaftor‐Ivacaftor
PEx: pulmonary exacerbation rate
ppFEV_1_
: percent predicted forced expiratory volume in 1 s
ppFVC: percent predicted forced vital capacity
PwCF: patients with cystic fibrosis
SwCl: sweat chloride concentration

## 1 Introduction

Cystic fibrosis (CF) is a rare hereditary genetic disease caused by defects in the cystic fibrosis transmembrane conductance regulator (CFTR) protein, which is involved in the regulation of hydro‐electrolyte exchange [1]. Defective CFTR protein causes the production of thick fluid secretions clogging the pancreatic and hepatic ducts. Furthermore, the pulmonary inflammation promotes the proliferation of pathogens, including *Staphylococcus aureus* and *Pseudomonas aeruginosa* , thereby increasing the risk of respiratory infections [2]. However, CF is a multisystem disease that affects various organs, resulting in pancreatic insufficiency, gut obstruction, biliary liver disease, and male infertility [3]. Until recently, medical treatments for cystic fibrosis focused mainly on symptom management. A triple combination of Elexacaftor‐Tezacaftor‐Ivacaftor (ETI) was approved in 2019 for the treatment of patients with cystic fibrosis (PwCF) carrying one or two copies of the Phe508del mutation [4]. ETI has demonstrated the ability to increase the percent predicted forced expiratory volume in 1 s (ppFEV_1_), improve respiratory symptoms, and decrease sweat chloride concentration in randomized phase 3 clinical trials [4, 5].

Nevertheless, the clinical trials were relatively short, with highly restrictive eligibility criteria, and long‐term outcomes such as mortality, lung transplantation, and adverse reactions could not be clearly determined [6]. Furthermore, to our knowledge, the most of studies had a limited number of patients [7]. Therefore, it is crucial to carry out observational studies, examining a larger and more heterogeneous population, including subjects typically excluded from clinical trials, such as patients with advanced lung disease. The response to ETI may differ between homozygotes and heterozygotes, as well as between naïve patients and those previously treated with other CFTR modulators. This is essential for achieving a deeper understanding of the disease in real‐world clinical practice while incorporating insights from clinical trials to enhance patient care.

Hence, the purpose of this study was to evaluate the effectiveness and safety of ETI in a large and heterogeneous cohort of PwCF and in these patient subgroups.

## 2 Material and Methods

This observational, monocentric study retrospectively reviewed data from 139 patients on ETI therapy followed at the CF Regional Reference Centre, Umberto I Hospital, Sapienza University of Rome, Italy. The study identified all eligible patients according to current national legislation. All participants involved in the study were aged 12 years or older at baseline with a confirmed diagnosis of CF. Each participant was either homozygous (Phe508del/Phe508del) or compound heterozygous (Phe508del/any, CF patients with two different mutated alleles) for the Phe508del mutation, as shown in Figure S1.

The study was approved by the local ethics committee (reference number 7096) and performed in accordance with the Declaration of Helsinki. Written informed consent was obtained from all study participants or their legal custodians prior to study inclusion.

The primary outcome was to assess the absolute change in ppFEV_1_ and the percent predicted forced vital capacity (ppFVC) at 6 (T6), 12 (T12) and 24 (T24) months from baseline (T0) in patients who received ETI. Lung function (ppFEV_1_ and ppFVC) was assessed by spirometry in accordance with the technical standards of the American Thoracic Society (ATS) and the European Respiratory Society (ERS) [8]. Furthermore, the assessment of respiratory function was conducted based on genotype (homozygous or compound heterozygous for Phe508del CFTR), differentiating between naïve subjects and patients previously treated with other CFTR modulators. The study also analyzed improvements in lung function among individuals with different degrees of lung impairment, including severe impairment (ppFEV_1_ < 40%), moderate impairment (40% < ppFEV_1_ < 70%) and normal/mild lung function (ppFEV_1_ > 70%) [8]. The occurrence of the pulmonary exacerbation rate (PEx) was identified as hospitalization or the need for intravenous antibiotic treatment in the previous year or after the start of CFTR modulator treatment. PEx data were collected for 12 months before and after starting treatment, according to clinical standards for the management of cystic fibrosis [9]. Baseline lung function, including ppFEV_1_ and ppFVC, was obtained from the last spirometric measurement taken before the start of therapy.

Secondary outcomes aimed to assess the impact of ETI treatment on BMI and sweat chloride concentration (SwCl) levels measured at 6 (T6), 12 (T12) and 24 (T24) months from baseline (T0). BMI is defined as the body mass divided by the square of the body height, and is expressed in units of kg/m^2^, resulting from mass in kilograms (kg) and height in meters (m). Patients' weight and height were measured using electronic scales and calibrated statimeters according to standard procedures described in a systematic review and meta‐analysis [10]. Sweat was collected using the quantitative pilocarpine iontophoresis test (QPIT), which is considered the gold standard for the diagnosis of CF. Chloride concentration was measured using a digital chloridometer [11]. Anonymous questionnaires from ETI‐treated patients were analyzed; the questionnaire is the Cystic Fibrosis Questionnaire‐Revised (CFQ‐R), which is regularly given to patients treated at each CF center as an additional tool for disease monitoring. CFQ‐R data were recorded at 12 and 24 months after the initiation of ETI treatment: normalized scores range from 0 to 100, with higher scores indicating higher patient‐reported quality of life. Other secondary endpoints included adverse events, microbial colonization, mortality, and transplantation rate.

### 2.1 Data Collection

Retrospective data collection took place from July 2021 to March 2024, covering patients who started ETI treatment during this period. We included patients with ETI prescriptions who were followed at our hospital for at least 6 months.

- Inclusion criteria: patients ≥ 12 years with a confirmed CF diagnosis, carrying at least one F508del allele, and receiving ETI therapy.
- Exclusion criteria: patients < 12 years, pregnancy, previous organ transplantation, severe comorbidities precluding follow‐up, and incomplete medical records.

Clinical and analytical data were analyzed from electronic medical records. The following demographic and clinical data were collected: sex, age, BMI, CFTR mutation genotype, prescription start date, comorbidities, lung function (ppFEV_1_, ppFVC), SwCl levels, pancreatic status, bronchial bacterial colonization, hospitalization for any cause, and request for intravenous antibiotic treatment. Data on treatment duration, dose changes, discontinuation, treatment‐related adverse events, mortality, and lung transplantation were recorded. Baseline measurements of these clinical parameters were obtained from the last quarter before the initiation of therapy.

### 2.2 Statistical Analysis

A descriptive analysis of the data was conducted, and an ANOVA (Analysis of Variance) was employed to evaluate the variance of ppFEV_1_, ppFVC, BMI, SwCl, and CFQ‐R. To support the analysis, scatter plots were performed for each variable mentioned, allowing for a visual assessment of the relationships and distributions. All tests were 2‐sided, accepting *p* < 0.05 as statistically significant, and confidence intervals were calculated at the 95% level. All analyses were performed using R software (R Core Team 2022). R: A language and environment for statistical computing. R Foundation for Statistical Computing, Vienna, Austria. URL: https://www.R‐project.org.

## 3 Results

A total of 163 subjects with CF were selected; 24 were excluded due to missing data at follow‐up. A total of 139 patients were included in the study (Table 1). Of the 139 subjects, 122 were adults (81.9%) and 17 were aged 12–17 (11.4%). Furthermore, three patients were on the waiting list for lung transplantation, and five eligible patients were being evaluated for inclusion on the transplant waiting list. The presented study reported a median follow‐up rate of 55.4% for patients who attended more than 50% of their scheduled visits at 24 months. It was therefore deduced that the median follow‐up period would be longer than 24 months. Therefore, a 24‐month follow‐up period was set up to ensure the collection and analysis of more robust data.

**TABLE 1:** Demographic and clinical characteristics of the patients at baseline.

Characteristic	*N* = 139
Female sex—*n* (%)	69 (49.6%)
Age in years‐mean (IQR)	30 (23–43)
ppFEV_1_, mean (IQR)	64% (23–124)
ppFVC, mean (IQR)	85% (35–153)
BMI, mean (IQR)	21.6 kg/m^2^ (15.2–38.9)
SwCL, mean (IQR)	84 mmol/L (38–194)
CFQ‐R, mean (IQR)	74% (65–80)
Genotype—*n* (%)	
Homozygous F508del	102 (73.4%)
Compound heterozygous F508del	37 (26.6%)
Comorbidity—*n* (%)	
Diabetes	23 (16.5%)
Pancreatic insufficiency	119 (85.6%)
CFTR modulator history—*n* (%)	
Naïve	102 (73.4%)
Lumacaftor + Ivacaftor	28 (20.1%)
Ivacaftor	1 (0.7%)
Tezacaftor + Ivacaftor	8 (5.8%)
Pulmonary exacerbation rate—*n* (%)	96 (69.1%)
Bacteriological infection—*n* (%)	
Bacterial colonization	138 (99.3%)
*Haemophilus influenzae*	19 (13.7%)
*Pseudomonas putida*	7 (5.0%)
*Pseudomonas A. rugose*	101 (72.7%)
*Pseudomonas A. mucoid*	83 (59.7%)
*Klebsiella pneumoniae*	13 (9.4%)
*Staphylococcus aureus*	117 (85%)
*Stenotrophomonas maltophilia*	26 (18.7%)
*Burkholderia cepacia*	8 (5.8%)

*Note:* Values are mean (with or without Interquartile range) or number of patients (percentage). Cystic fibrosis transmembrane conductance regulator (CFTR). Percent predicted forced expiratory volume in 1 s (ppFEV_1_), percent predicted forced vital capacity (ppFVC), body‐mass index (BMI), sweat chloride concentration (SwCl), CFQ‐R respiratory domain score: normalized scores range from 0 to 100 with higher scores indicating higher patient‐reported quality of life.

### 3.1 Pulmonary Function

**FIGURE 1:**
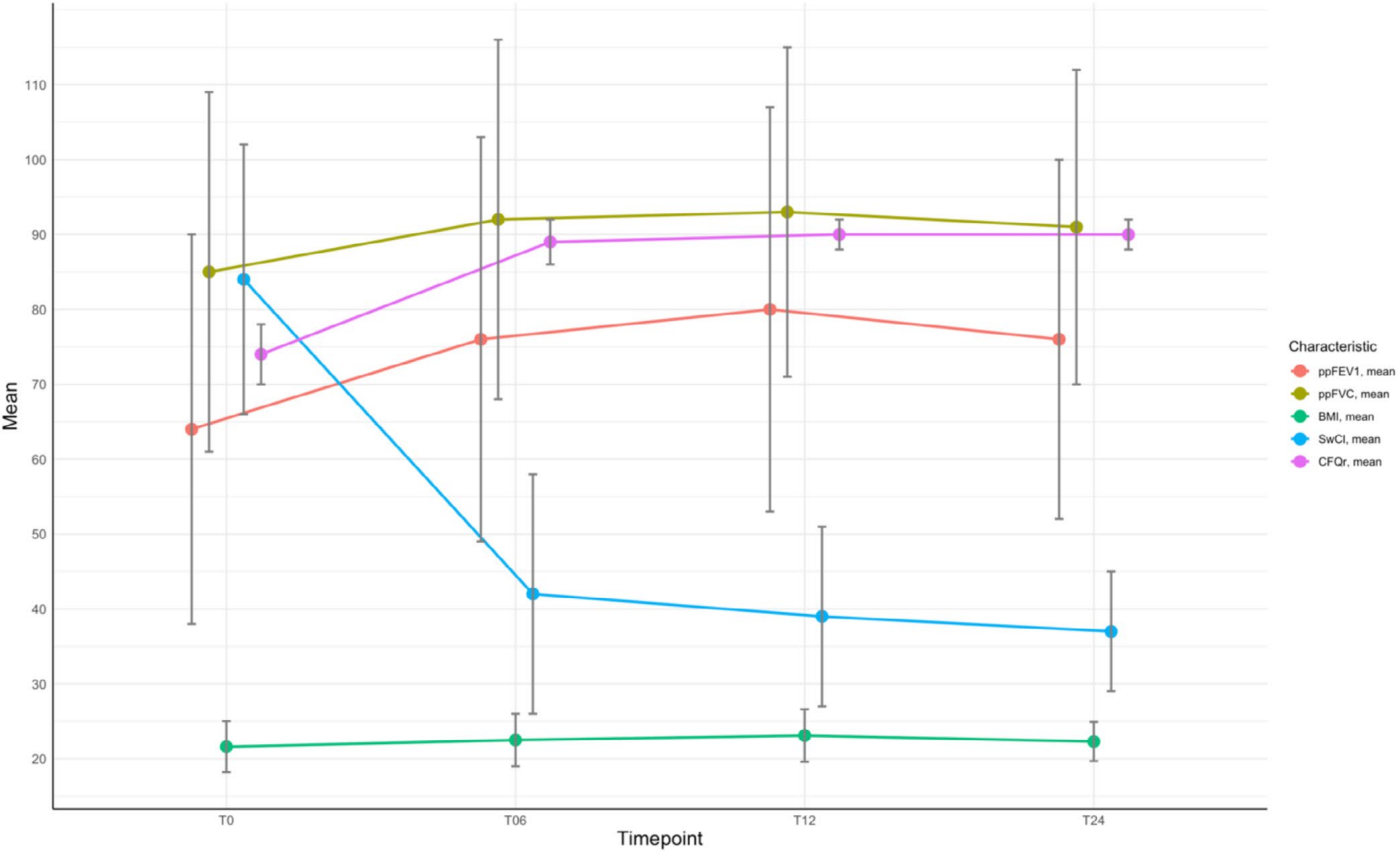
The figure illustrates the absolute change from baseline for each clinical characteristic: Percent predicted forced expiratory volume in 1 s (ppFEV_1_), percent predicted forced vital capacity (ppFVC), body‐mass index (BMI), sweat chloride concentration (SwCl), CFQ‐R respiratory domain score measured at T0 (baseline), T6 (after 6 months of ETI), T12 (after 12 months of ETI), and T24 (after 24 months of ETI).

The overall improvement in lung function in patients treated with ETI is illustrated in Table 2 and Figure 1. ETI showed a significant increase in the absolute change in ppFEV_1_ of 12% at 6 months compared to baseline, 16% at 12 months, and 12% after 2 years of therapy (*p* < 0.001). Furthermore, ETI had a positive effect on ppFVC, with an absolute change at 6, 12, and 24 months from baseline of 7%, 8%, and 6%, respectively (*p* = 0.044).

**TABLE 2:** Absolute change from baseline in percent predicted forced expiratory volume in 1 s (ppFEV_1_), percent predicted forced vital capacity (ppFVC), body‐mass index (BMI), sweat chloride concentration (SwCl) and CFQ‐R respiratory domain score.

Clinical outcomes	Timepoint				*p* value^a^
	T0	T6	T12	T24	
ppFEV_1_, mean (SD)	64 (26)	76 (27)	80 (27)	76 (24)	**< 0.001**
ppFVC, mean (SD)	85 (24)	92 (24)	93 (22)	91 (21)	**0.044**
BMI, mean (SD)	21.6 (3.4)	22.5 (3.5)	23.1 (3.5)	22.3 (2.6)	**0.005**
SwCL, mean (SD)	84 (18)	42 (16)	39 (12)	37 (8)	**< 0.001**
CFQ‐R, mean (SD)	74 (4)	89 (3)	90 (2)	90 (2)	**< 0.001**

*Note:* The values shown in bold indicate statistically significant results (*p* < 0.05).

Abbreviations: T0 = baseline, T6 = after 6 months of treatment, T12 = after 12 months of treatment, T24 = after 24 months of treatment.

^a^ One‐way ANOVA.

### 3.2 Pulmonary Severity Analysis

**FIGURE 2:**
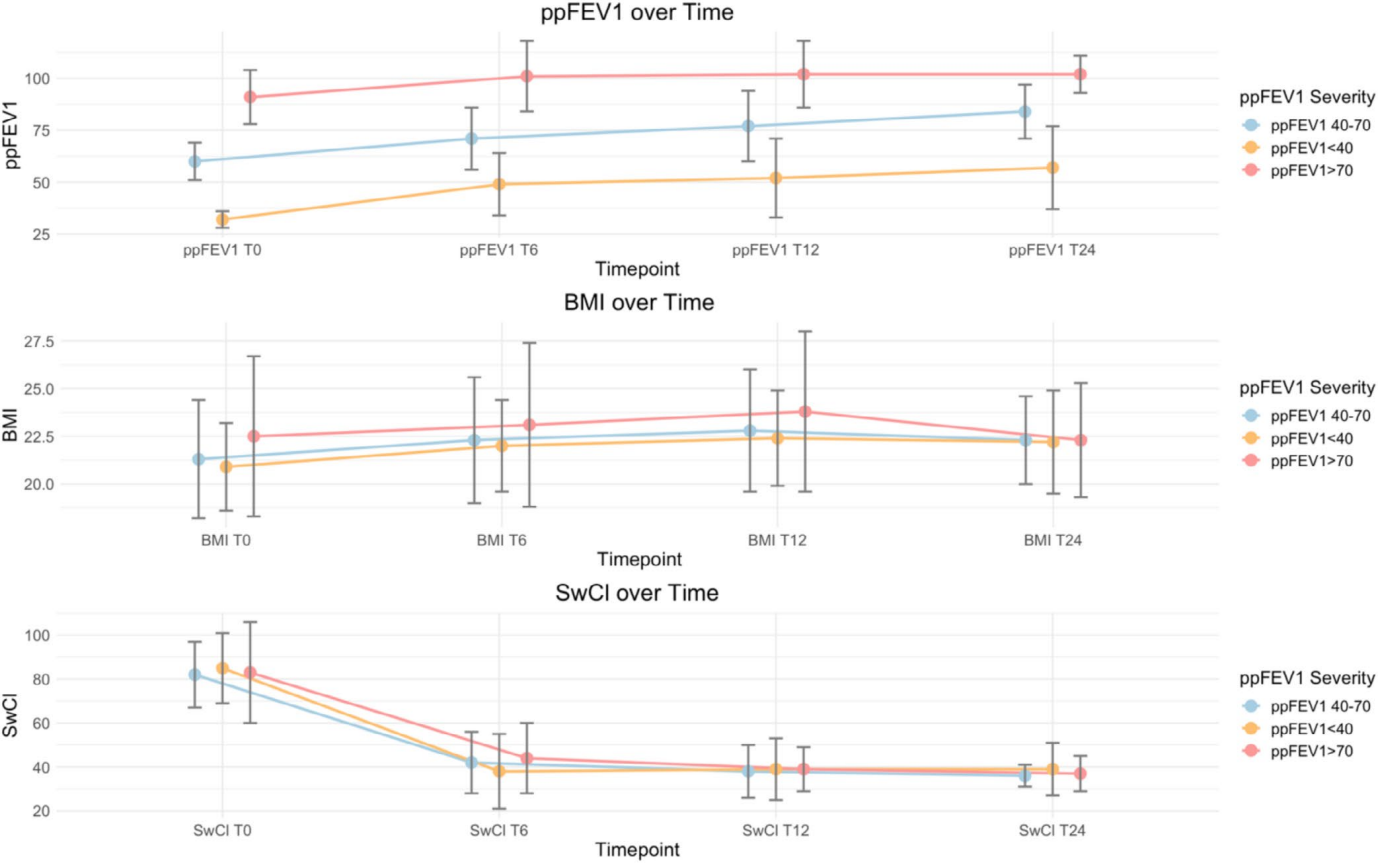
The figure illustrates the absolute change from baseline in percent predicted forced expiratory volume in 1 s (ppFEV_1_), body‐mass index (BMI), sweat chloride concentration (SwCl), according to ppFEV_1_ severity, measured at T0 (baseline), T6 (after 6 months of ETI), T12 (after 12 months of ETI), and T24 (after 24 months of ETI).

40 subjects were ppFEV_1_ < 40% at baseline, 44 patients were ppFEV_1_ between 40% and 70%, and 55 subjects were ppFEV_1_ > 70% (Table 3, Figure 2). The subgroup of subjects with ppFEV_1_ < 40% showed an absolute change of 17% after 6 months of ETI therapy, with an improvement in values to 20% at 12 months and 25% at 24 months (*p* < 0.001). The study results demonstrated a significant increase in ppFEV_1_ in patients with severe pulmonary impairment (ppFEV_1_ < 40). The initial cohort of 40 patients with ppFEV_1_ < 40% at baseline was reduced to only 10 patients after 12 months of therapy. Moreover, 90.9% of these patients exhibited a *P. aeruginosa* (Rugose and Mucoid) colonization. Patients with ppFEV_1_ between 40% and 70% showed a significant and progressive increase in ppFEV_1_ of 11%, 17%, and 24% at 6, 12, and 24 months (*p* < 0.001). Finally, patients with a ppFEV_1_ greater than 70% demonstrated a mean increase of 10% at 6 months, with an 11% increase observed at 12 months in comparison to the baseline (*p* < 0.001).

**TABLE 3:** Absolute change from baseline in percent predicted forced expiratory volume in 1 s (ppFEV_1_), body‐mass index (BMI), and sweat chloride concentration (SwCl) according to ppFEV_1_ severity (a), genotype (b), and CFTR modulator clinical history (c).

	Timepoint									
	(a) ppFEV_1_ < 40, *N* = 40	40 < ppFEV_1_ < 70, *N* = 44	ppFEV_1_ > 70, *N* = 55	*p* value^a^	(b) Homozygotes, *N* = 102	Heterozygotes, *N* = 37	*p* value^a^	(c) Naive, *N* = 102	Previously treated, *N* = 37	*p* value^a^
ppFEV_1_										
T0, mean (SD)	32 (4)	60 (9)	91 (13)	**< 0.001**	70 (26)	49 (21)	**< 0.001**	64 (26)	64 (27)	0.90
T6, mean (SD)	49 (15)	71 (15)	101 (17)	**< 0.001**	80 (27)	67 (23)	**0.011**	77 (26)	74 (28)	0.51
T12, mean (SD)	52 (19)	77 (17)	102 (16)	**< 0.001**	83 (27)	73 (24)	0.078	81 (26)	77 (28)	0.44
T24, mean (SD)	57 (20)	84 (13)	102 (9)	**< 0.001**	76 (25)	75 (22)	0.85	79 (22)	68 (25)	**0.005**
BMI										
T0, mean (SD)	20.9 (2.3)	21.3 (3.1)	22.5 (4.2)	0.05	22.1 (3.6)	20.3 (2.4)	**0.004**	21.5 (3.6)	22.0 (3.2)	0.43
T6, mean (SD)	22.0 (2.4)	22.3 (3.3)	23.1 (4.3)	0.28	23.0 (3.7)	21.3 (2.4)	**0.010**	22.5 (3.7)	22.7 (3.1)	0.77
T12, mean (SD)	22.4 (2.5)	22.8 (3.2)	23.8 (4.2)	0.14	23.6 (3.7)	21.7 (2.3)	**0.005**	23.0 (3.5)	23.4 (3.5)	0.61
T24, mean (SD)	22.2 (2.7)	22.3 (2.3)	22.3 (3.0)	0.98	22.4 (2.7)	22.0 (2.5)	0.55	22.2 (2.4)	22.5 (3.1)	0.62
SwCl										
T0, mean (SD)	85 (16)	82 (15)	83 (23)	0.74	83 (19)	87 (16)	0.27	86 (20)	78 (11)	**0.020**
T6, mean (SD)	38 (17)	42 (14)	44 (16)	0.21	41 (16)	44 (15)	0.43	43 (16)	38 (16)	0.12
T12, mean (SD)	39 (14)	38 (12)	39 (10)	0.90	37 (10)	38 (10)	**0.004**	40 (12)	34 (11)	**0.010**
T24, mean (SD)	39 (12)	36 (5)	37 (8)	0.68	37 (7)	38 (10)	0.45	38 (9)	36 (8)	0.52

*Note:* The values shown in bold indicate statistically significant results (*p* < 0.05).

Abbreviations: T0 = baseline, T6 = after 6 months of treatment, T12 = after 12 months of treatment, T24 = after 24 months of treatment.

^a^ One‐way ANOVA.

### 3.3 Genotype Analysis

**FIGURE 3:**
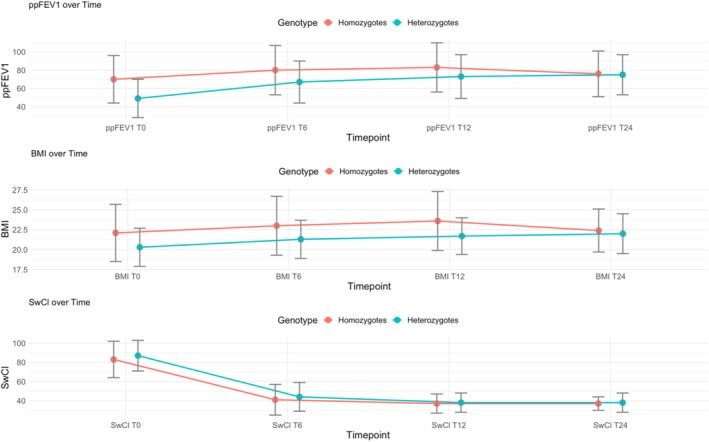
The figure illustrates the absolute change from baseline in percent predicted forced expiratory volume in 1 s (ppFEV_1_), body‐mass index (BMI), sweat chloride concentration (SwCl), according to genotype, measured at T0 (baseline), T6 (after 6 months of ETI), T12 (after 12 months of ETI), and T24 (after 24 months of ETI).

The overall patients, regardless of whether they were in the compound heterozygous or homozygous Phe508del group, showed a comparable increase in ppFEV_1_ from baseline during the follow‐up period, with values stabilizing at 24 months (Table 3, Figure 3). Statistical analysis comparing the two groups showed that the compound heterozygous group started with a significantly lower ppFEV_1_ value at baseline (*p* < 0.001) and ultimately achieved an increase of 26%. In addition, the homozygous group, which had a better initial clinical status with a baseline ppFEV_1_ greater than 70%, showed an improvement of 10% at 6 months and 13% at 12 months, with values remaining stable at 6% after 24 months of therapy.

### 3.4 Response Analysis in Patients Previously Treated With Another CFTR Modulator

**FIGURE 4:**
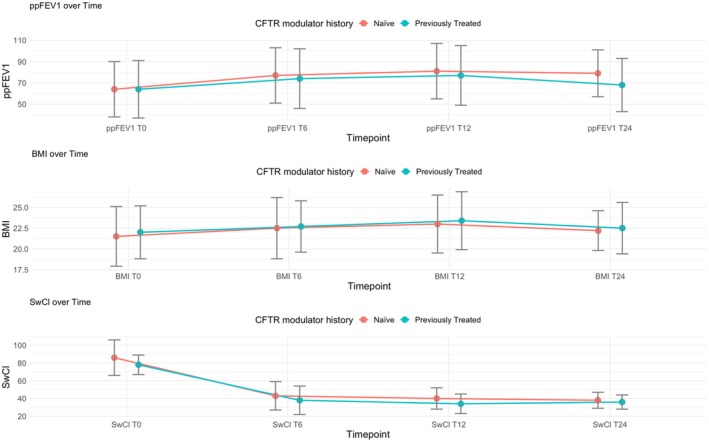
The figure illustrates the absolute change from baseline in percent predicted forced expiratory volume in 1 s (ppFEV_1_), body‐mass index (BMI), sweat chloride concentration (SwCl), according to CFTR modulator history, measured at T0 (baseline), T6 (after 6 months of ETI), T12 (after 12 months of ETI), and T24 (after 24 months of ETI).

CFTR‐naïve patients showed a significant change in ppFEV_1_ within the first 6 months of therapy (13%). This value increased further in the follow‐up at 12 months, with an average change of 17% and stabilization at 15% after 24 months. However, patients previously treated with other CFTR modulators showed an unusual pattern in the change of ppFEV_1_: an increase of 10% at 6 months and 13% after 12 months, followed by a reduction of 4% after 2 years of ETI treatment. A statistically significant difference between the two groups was found at T24 (*p* = 0.005) (Table 3, Figure 4).

### 3.5 Body Mass Index

The nutritional status of 139 patients before starting ETI therapy was: 72% were normal weight, with an average BMI of 21.29 kg/m^2^. 13.7% were underweight, with an average BMI of 17.02 kg/m^2^. 12.2% were overweight, with an average BMI of 26.74 kg/m^2^, whereas 2.1% were obese, with an average BMI of 33.72 kg/m^2^. The average BMI was 21.6 kg/m^2^.

The triple combination therapy led to a significant improvement in BMI for all subjects, with an increase of 0.9 kg/m^2^ in the first 6 months of treatment. After 12 months, the BMI increased by 1.5 kg/m^2^, but then decreased to 0.7 kg/m^2^ after 24 months (Table 2, Figure 1). However, there was no change in BMI for people with severe lung problems (Table 3, Figure 2). A subgroup analysis by CFTR genotype showed a significant change in BMI between homozygous and compound heterozygous individuals at 6 and 12 months, but not at 24 months (Table 3, Figure 3). Weight gain was slightly higher in CFTR‐naïve PwCF compared to those previously treated with another modulator after 6 months of therapy (1.0 vs. 0.7 kg/m^2^), with comparable increases in both groups at T12 (1.5 vs. 1.4 kg/m^2^) and subsequent decreases in both at T24 (0.7 vs. 0.55 kg/m^2^) (Table 3, Figure 4).

### 3.6 Pulmonary Exacerbation Rate

In the 12 months prior to ETI treatment, 69.1% (*N* = 96) of the patients required hospitalization or intravenous antibiotic administration. Among the overall patients (*N* = 80), 44 had at least one episode, 23 had at least two episodes, 7 had at least three episodes, and 6 had at least four episodes. However, 12 months after the ETI therapy was initiated, the frequency of infectious exacerbations had significantly decreased to 10.8% (*N* = 15). The triple combination therapy necessitated hospitalization or intravenous antibiotic treatment in 11 patients and more than two episodes per year in 4 patients within the 12 months after the initiation of ETI treatment (Figure S2).

### 3.7 Sweat Chloride Concentration Test

SwCl overall reduction was observed, with an initial mean value of 84 mmol/L (±18) (Table 2, Figure 1). The clinical results showed a progressive decrease of SwCl in all patients treated with ETI, with an average reduction to 42 mmol/L (±16) at 6 months and 39 mmol/L (±12) at 12 months. This value subsequently decreased to 37 mmol/L (±8) at 24 months after the initiation of ETI therapy (*p* < 0.001). No statistically significant variation was observed in patients with severe respiratory impairment (Table 3, Figure 2). Furthermore, in a stratified analysis by CFTR modulator history, a statistically significant variation was observed after 12 months of ETI treatment (*p* = 0.010) (Table 3, Figure 4).

### 3.8 CFQ‐R Respiratory Domain Scores

The CFQ‐R scores of patients during treatment with ETI were analyzed (Table 2, Figure 1). The questionnaires showed that the respiratory domain of the CFQ‐R improved. After 6 months, there was a 15% increase from the baseline score. After 12 and 24 months, there was an additional average increase of 16 percentage points (*p* < 0.001).

### 3.9 Microbial Colonization

Analyses of sputum samples and cultures for common pathogens revealed that almost the entire study population (99.3%) exhibited microbial colonization at baseline (Table 1). The most prevalent strain identified in patients treated with ETI was *S. aureus* (85%), followed by mucosal phenotype Pseudomonas A. Rugose (73%), and Pseudomonas A. Mucoid (60%).

### 3.10 Transplantation Rates

Two patients on the transplant waiting list were removed from the list due to improvements in their lung function. After 24 months, the two patients (with an average ppFEV_1_ value of 28.5 at baseline) exhibited a 9% increase in their ppFEV_1_ value. The five patients on the transplant waiting list were also monitored. The patients began with an average baseline ppFEV_1_ value of 27.2, which increased by 5.8%, 12%, and 13.8% at 6, 12, and 24‐month follow‐up periods, respectively. These patients were excluded from the transplant list because their lungs and overall health had improved, and they had fewer annual flare‐ups.

### 3.11 Safety

The most frequently reported adverse drug reactions (ADRs) were increases in creatine phosphokinase (CPK), occurring in 20.86% of cases (*N* = 29). Of these, 58.62% (*N* = 17) were of moderate to severe grade, while 41.38% (*N* = 12) were of mild grade. Furthermore, an elevation in bilirubin levels was observed in 5.75% (*N* = 8) of the study cohort, with three cases classified as mild and five cases as moderate in severity. Other ADRs included a case of cutaneous rash following ETI administration and a case of neuropsychiatric disorder in an individual with a history of psychiatric episodes and a predisposition to such conditions. All patients who experienced ADRs reduced their dosage but continued ETI therapy, which resulted in the resolution of symptoms. No patients treated with ETI died because of the treatment.

## 4 Discussion

ETI has been shown to be an effective treatment for PwCF, with a positive impact on disease control and quality of life. All parameters measured demonstrated significant improvements in lung function, BMI, SwCl, CFQ‐R respiratory domain scores, and the rate of pulmonary exacerbations within the first 6 months of therapy. The results demonstrated a significant and sustained improvement in lung function in all patients examined after 12 months of therapy (ppFEV_1_ = 16%, *p* < 0.001), with a slight decline in the values after 24 months.

The increase in ppFEV_1_ was more considerable than that observed in phase III clinical trials, which reported an absolute change in ppFEV_1_ of 10% in PwCF who were homozygous for the Phe508del mutation [4] and 13.8% in PwCF who were compound heterozygous for Phe508del [5]. Similar results were also recorded in the observational studies conducted by Burgel et al. (+15.1%) [12], Carnovale et al. (+14.2%) [13], Kos et al. (+13.7%) [14], and Savi et al. (+13%) [15]. The results of this study suggest the efficacy of ETI in improving lung function in PwCF. The study also examined the clinical benefits of ETI in PwCF with advanced lung disease. These patients are usually excluded from registrational clinical trials due to progressively worsening lung function. This subgroup of PwCF demonstrated a favorable response to ETI treatment, with an absolute increase in ppFEV_1_ of 17% after 6 months of therapy. This is comparable to the group of patients with ppFEV_1_ < 40 treated in the phase III study and an observational study [5, 6, 8, 9]. Following 12 months of therapy, the ppFEV_1_ of this subgroup of patients demonstrated a significant increase of 20%, which is higher than the values reported by Carnovale et al. [13], who found an absolute change in ppFEV_1_ of 14.48% after 1 year of ETI. PwCF treated with other CFTR modulators showed poor clinical conditions and modest changes in lung function. Initiation of ETI resulted in a rapid and significant improvement in lung function in all patients. Of particular interest is the change in response at 24 months in PwCF who were naïve to treatment with ETI (+15%) compared to those treated with other modulators (+4%) (*p* = 0.005). Our data are comparable with the findings of a large German study of over 2600 PwCF [16], which showed a greater increase in ppFEV_1_ in CFTR‐naïve subjects than in those who had previously taken a CFTR modulator [12.6% (95% CI 11.9–13.4) vs. 9.7% (95% CI 9.0–10.5)].

**FIGURE 5:**
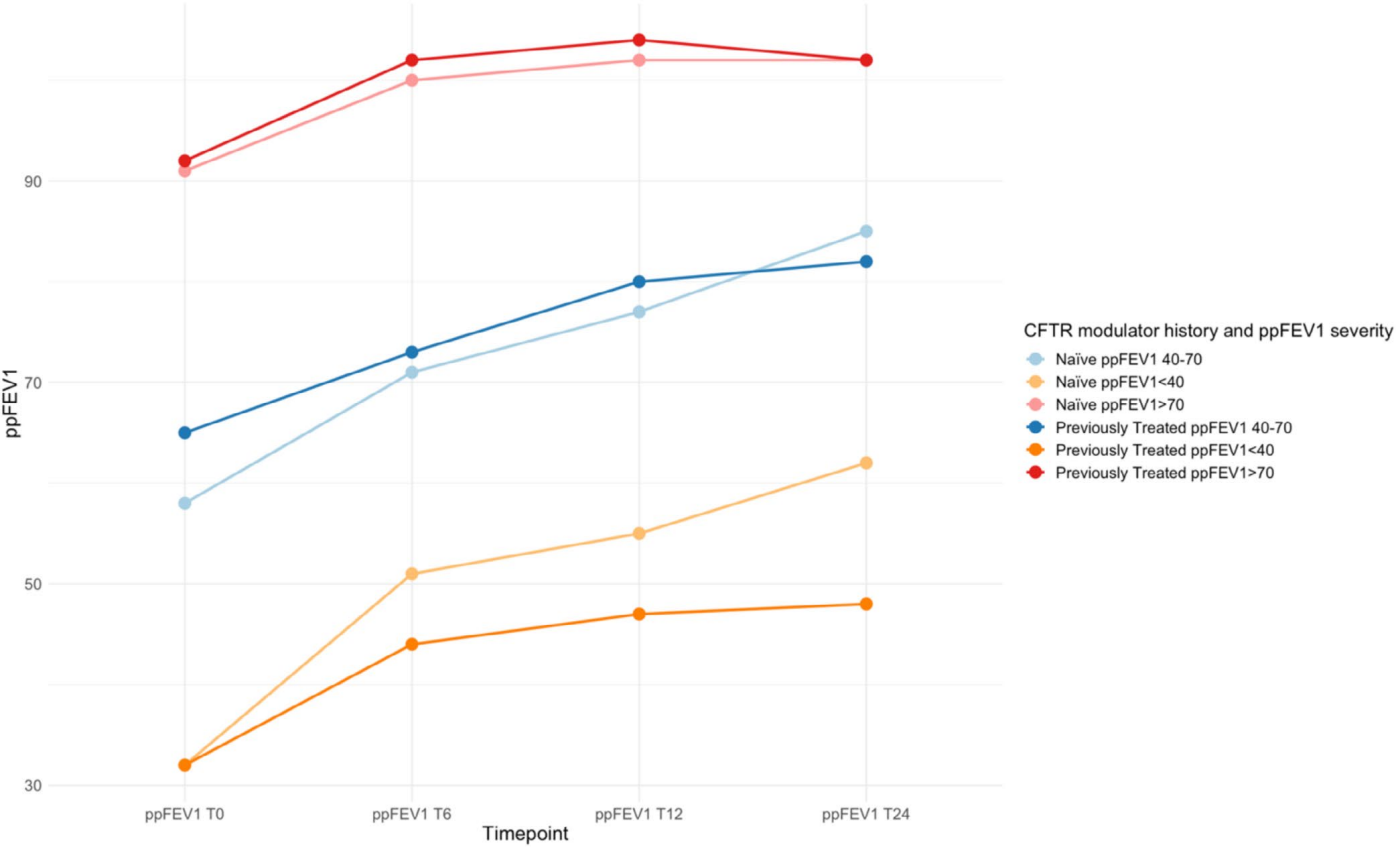
The figure illustrates the absolute change from baseline in percent predicted forced expiratory volume in 1 s (ppFEV_1_), according to CFTR modulator history and ppFEV_1_ severity, measured at T0 (baseline), T6 (after 6 months of ETI), T12 (after 12 months of ETI), and T24 (after 24 months of ETI).

In our study, subjects with ppFEV_1_ < 40 who were naïve to therapy showed a significant improvement in absolute change (+30%) 2 years after initiating ETI therapy. In contrast, previously treated patients with ppFEV_1_ < 40 who were receiving other modulators demonstrated a significant improvement (+16%), although this was not as pronounced as the results observed in CFTR‐naïve subjects (Figure 5). Overall, ETI therapy resulted in an increase in ppFEV_1_ during the study period for both the homozygous and compound heterozygous patient cohorts, consistent with several observational studies [10, 17] (Table 3, Figure 3). Although the two groups had different clinical conditions and baseline values that were not evenly distributed [49 (±21) for heterozygotes, 70 (±26) for homozygotes], they achieved similar ppFEV_1_ values after 24 months of therapy [75 (±22) for heterozygotes, 76 (±25) for homozygotes], supporting the efficacy of ETI in both genotypes.

The administration of ETI significantly reduced the number of PEx, accompanied by a statistically and clinically significant decline in the incidence of PEx that required hospitalization or intravenous antibiotic therapy. The study population exhibited a high frequency of pulmonary exacerbations in the year prior to the start of ETI therapy (0.9 PEx/year). A notable reduction in the incidence of PEx was observed in the year following ETI treatment, with a 58.3% reduction in frequency compared to the number of exacerbations prior to ETI treatment. However, the results of the PwCF who had at least two PEx after 12 months of ETI treatment may be biased, as some of these patients had poor compliance. These results can be compared to the phase III study in which a 63% reduction of exacerbations was observed in PwCF treated with ETI compared to the previous year [5]. This issue was regarded in different studies that showed that three exacerbations per year are critical in terms of lung function decline in CF. PwCF with more than two PEx per year have an increased risk of death or lung transplantation within 3 years [11, 18, 19].

Microbiological analysis of sputum samples revealed that the predominant colonizing strains were *S. aureus* and *P. aeruginosa* (mucoid and rugose phenotypes). However, despite the marked improvement in the reduction of pulmonary exacerbations, the proportion of PwCF reporting persistent or intermittent bacterial colonization remained high. Recent studies showed that improvements in clinical status and the efficacy of ETI may be compromised by persistent infections, probably associated with antibiotic resistance mechanisms [14, 20].

In the present study, we observed a progressive improvement in BMI in all subjects, with an average change of +1.5 kg/m^2^ after 12 months of therapy compared to baseline. This sustained increase in BMI is consistent with the findings of several observational studies [10, 11, 21]. However, a decrease in BMI was observed at the 24‐month follow‐up. Potential explanations for the observed phenomenon may include the establishment of tolerance to ETI or a change in eating habits due to an overall improvement in the patient's clinical condition, which may have altered the nutritional plan. No statistically significant weight gain was observed in PwCF naïve compared to patients previously treated with another CFTR modulator after 24 months of ETI (*p* = 0.62), contrary to the results of the study by Burgel et al. [12]. Furthermore, stratifying by genotype, compound heterozygous subjects showed a mean BMI change of 1.4 kg/m^2^ after 12 months of therapy, with a further increase to 1.75 kg/m^2^ after 2 years. In the homozygous group, a mean increase in BMI of 1.5 kg/m^2^ was observed following 1‐year treatment with ETI, with values fluctuating up to 0.34 kg/m^2^ at 24 months before stabilizing at baseline. Consequently, there was no significant improvement in nutritional status in the homozygous group.

In agreement with the literature [12, 22], SwCl decreased significantly by 42 mmol/L after 6 months of ETI in all subjects, including Phe508del homozygotes. At subsequent follow‐up, the reduction remained constant. These results are consistent with phase III clinical trials [5]. Therefore, we are in agreement with the authors that the marked reduction in SwCl reflects a significant improvement in CFTR functionality in all ETI‐treated subjects. CFQ‐R score improved by 16 points (*p* < 0.001) to a final score of 90 points after 24 months of treatment. The mean score difference achieved after 24 months of treatment was comparable to that observed in an observational study [15], and the final overall score was consistent with results from a phase III clinical trial [5].

Overall, our findings suggest genotype‐independent effects, although statistical differences were present in certain cases. Specifically, the compound heterozygous group had a significantly lower baseline ppFEV_1_ (*p* < 0.001) and showed a greater percentage improvement over time. BMI differences were significant at 6 and 12 months but not at 24 months, and sweat chloride reduction showed statistical variation after 12 months (*p* = 0.010).

ETI therapy was well tolerated, with no participants discontinuing treatment during the 2‐year follow‐up period. These results are consistent with phase III studies in homozygous and compound heterozygous Phe508del patients, which reported discontinuation rates of 0% and 1%, respectively [4, 5]. There were no deaths during the study period analyzed. 28% of patients experienced adverse events, which resolved rapidly with a reduction in the daily dose. ADRs observed included increases in CPK (20.86%) and bilirubin (5.75%). One patient experienced a mild rash following ETI administration. After dose reduction, the triple combination was well tolerated, and no further episodes occurred. In addition, a patient with a predisposition to neuropsychiatric disorders experienced episodes of amnesia with learning and behavioral difficulties. However, the patient continued to receive ETI therapy, with a reduction in the daily dose and no further manifestations. It has been hypothesized that neuropsychiatric disorders are driven by the modulation of CFTR and chloride transport within the brain, resulting in altered GABA function [23]. Furthermore, the elevation of several pro‐inflammatory factors, including IL‐6 and CRP, has been linked to the development of neuropsychiatric disorders through the activation of glial cells. This pathway may have exacerbated a pre‐existing clinical condition in a patient with elevated baseline inflammation, although the relationship between ETI and adverse events is uncertain [24]. Consequently, this potential association requires further investigation.

Notable clinical improvements were observed in all patients awaiting lung transplantation, as well as in the five eligible patients evaluated for listing. This improvement led to a significant increase in life expectancy, as it was no longer indicated to proceed with transplantation. Results are consistent with those of several observational studies [9, 25, 26]. The observed improvement was sufficiently pronounced to influence the clinical conditions of patients in terms of lung transplant planning in accordance with guidelines in CF [27].

The strengths of the study are the sample size and the length of follow‐up, which provided important safety data over a long observation period. Nevertheless, it is important to consider the limitations of the study. The retrospective nature of the study presents certain limitations, including the potential for selection bias and difficulty in measuring treatment effects, given that patients were not followed in real time. Moreover, the current study may be limited by the specific nature of the study population, including a monocentric experience and the absence of a control group. To address these issues, we stratified the overall population according to the severity of ppFEV_1_. Despite our efforts to include a larger number of eligible subjects, only 55.4% of patients completed the 2‐year follow‐up period following the initiation of ETI treatment.

Possible mechanisms underlying the decline in ppFEV_1_ and BMI at 24 months include potential drug tolerance, lifestyle modifications, and long‐term disease progression. Several hypotheses may explain this trend: (i) a partial loss of CFTR modulatory efficacy over time due to adaptive cellular responses or reduced drug adherence; (ii) an altered metabolic state that influences weight gain and BMI stabilization; and (iii) the progressive nature of cystic fibrosis, which may counterbalance some of the initial therapeutic benefits. While ETI provides significant clinical benefits, these findings underscore the importance of long‐term monitoring and potential adjunctive strategies to sustain clinical improvements beyond 24 months [28].

Although CFTR function recovery is necessary to restore all clinical parameters, it may not be sufficient. CFTR restoration through modulatory therapy addresses the primary molecular defect but does not directly reverse the long‐term structural lung damage, chronic inflammation, or entrenched bacterial colonization that have developed over years of disease progression. This highlights the need for a multimodal therapeutic approach that includes antimicrobial strategies, anti‐inflammatory agents, and close monitoring of disease progression. Further research should focus on identifying complementary treatments that optimize the benefits of CFTR modulation and ensure sustained improvements across all clinical parameters.

In conclusion, this real‐world study provided important insights into the clinical impact of ETI therapy in a large and heterogeneous cohort of PwCF. Our findings demonstrated a significant and sustained improvement in lung function in patients after 24 months of ETI. Furthermore, the safety data demonstrated an acceptable ADRs and tolerability profile, which did not result in treatment discontinuation. However, PEx remains a significant challenge due to the continued prevalence of chronic or recurrent infections. In order to gain a better understanding of the long‐term effects of ETI, it is essential to extend the observation period and to include a larger cohort of PwCF in future multicenter studies.

## Author Contributions

**Nicola Perrotta:** writing – review and editing, writing – original draft, visualization, validation, supervision, project administration, methodology, investigation, formal analysis, conceptualization. **Luigi Angelo Fiorito:** writing – review and editing, writing – original draft, visualization, supervision, project administration, methodology, investigation, conceptualization. **Gianfranco Casini:** writing – review and editing, methodology, formal analysis, data curation, conceptualization. **Rossella Gentile:** software, methodology, formal analysis, data curation, conceptualization. **Roberta Vescovo:** software, resources, methodology, formal analysis, data curation, conceptualization. **Alfonso Piciocchi:** software, methodology, formal analysis, data curation, conceptualization. **Roberta Lobello:** supervision. **Carlo Cappelli:** supervision, conceptualization. **Roberto Poscia:** validation, supervision. **Giuseppe Cimino:** visualization, validation, supervision, methodology, investigation, conceptualization.

## Ethics Statement

This study was reviewed and approved by the Ethics Committee of Sapienza, University of Rome, with the approval number: 7096. All patients provided written informed consent to participate in the study and for their data to be published. All data collected was treated in accordance with current privacy regulations and Good Clinical Practice (GCP). Data were collected anonymously; each patient was assigned an identification code.

## Conflicts of Interest

The authors declare no conflicts of interest.

## Supporting information


Figure S1.

Figure S2.

## Data Availability

The data that support the findings of this study are available on request from the corresponding author and are not publicly available due to privacy or ethical restrictions.
