# Supplementary material for: Improved Clinical Outcomes With Elexacaftor/Tezacaftor/Ivacaftor in Patients With Cystic Fibrosis and Advanced Lung Disease: Real‐World Evidence From an Italian Single‐Center Study
Source: Pharmacol Res Perspect. 2025 Apr 2;13(2):e70083. doi: 10.1002/prp2.70083 (PMC11965699; doi:10.1002/prp2.70083)
Supplement: Supplementary file 1 — Figure S1. [file PRP2-13-e70083-s002.docx]

| **Variant legacy name** | **Variant cDNA name** | **hgvs_genomic_grch38** | **N** | **%** |
| --- | --- | --- | --- | --- |
| 1259insA | c.1130dup | NC_000007.14:g.117542029dup | 1 | 2.7 |
| 1717-1G ->A | c.1585-1G>A | NC_000007.14:g.117587738G>A | 2 | 5.4 |
| 2789+5G->A | c.2657+2_2657+3insA | NC_000007.14:g.117602865_117602866insA | 1 | 2.7 |
| 621+1G->A | c.489+1G>A | NC_000007.14:g.117531115G>A | 2 | 5.4 |
| 711+1G->T | c.579+1G>T | NC_000007.14:g.117534366G>T | 1 | 2.7 |
| 991del5 | c.861_865del | NC_000007.14:g.117536665_117536669del | 1 | 2.7 |
| CFTRdele14b-17b | c.(2619+1_2620-1)_(3367+1_3368-1)del | structural variant; genomic coordinates not provided | 1 | 2.7 |
| G542X | c.1624G>T | NC_000007.14:g.117587778G>T | 3 | 8.1 |
| G85E | c.254G>A | NC_000007.14:g.117509123G>A | 2 | 5.4 |
| L1065P | c.3194T>C | NC_000007.14:g.117611635T>C | 1 | 2.7 |
| L1077P | c.3230T>C | NC_000007.14:g.117611671T>C | 4 | 10.8 |
| N1303K | c.3909C>G | NC_000007.14:g.117652877C>G | 10 | 27.0 |
| R347P | c.1040G>C | NC_000007.14:g.117540270G>C | 1 | 2.7 |
| R553X | c.1657C>T | NC_000007.14:g.117587811C>T | 2 | 5.4 |
| W1282X | c.3846G>A | NC_000007.14:g.117642566G>A | 4 | 10.8 |
| W19X | c.56G>A | NC_000007.14:g.117504255G>A | 1 | 2.7 |

Figure S1: Classification of compound heterozygous genotypes: listing the specific second pathogenic variants of the 37 compound heterozygous patients
