# Supplementary material for: Improved Clinical Outcomes With Elexacaftor/Tezacaftor/Ivacaftor in Patients With Cystic Fibrosis and Advanced Lung Disease: Real‐World Evidence From an Italian Single‐Center Study
Source: Pharmacol Res Perspect. 2025 Apr 2;13(2):e70083. doi: 10.1002/prp2.70083 (PMC11965699; doi:10.1002/prp2.70083)
Supplement: Supplementary file 2 — Figure S2. [file PRP2-13-e70083-s001.docx]

Supplementary Figure S2: PEx pre- and post-ETI. Number of care episodes of PEx requiring admission or administration of IV antibiotic therapy in the previous 12 months at the start of ETI and after 12 months of ETI. Eighty patients had episodes of PEx prior to ETI: 44 had one episode [blue], 23 had two episodes [yellow], 7 had three episodes [orange] and 6 had four episodes [red]. The frequency of infectious exacerbations had significantly decreased to 10.8% (n=15) 12 months after ETI: 11 patients had one episode [blue] and 4 had two episodes [yellow].
